# Preparation and Application of Shen Ling Cao Composite Particles with Different Structures Based on Co-Spray Drying

**DOI:** 10.3390/ph18091369

**Published:** 2025-09-12

**Authors:** Zhe Li, Caiyun Sun, Ping Sun, Lingyu Yang, Qi Yang, Weifeng Zhu, Yongmei Guan, Wenjun Liu, Liangshan Ming

**Affiliations:** 1 Key Laboratory of Modern Preparation of TCM, Ministry of Education, Institute for Advanced Study, Jiangxi University of Chinese Medicine, Nanchang 330004, China; lizhezd@163.com (Z.L.); 18648193560@163.com (C.S.); zwf0322@126.com (W.Z.); guanym2008@163.com (Y.G.); 2 Jiangzhong Pharmaceutical Co., Ltd., Nanchang 330049, China; sp@crjz.com (P.S.); yly@crjz.com (L.Y.); yangqi@crjz.com (Q.Y.); 3 State Key Laboratory for the Modernization of Classical and Famous Prescriptions of Chinese Medicine, Nanchang 330096, China

**Keywords:** direct compaction, particle engineering, flowability, compactibility, co-spray drying

## Abstract

**Objectives:** Sen Ling Cao (SLC) is an excellent health food that has health-promoting functions, such as alleviating physical fatigue and boosting immune function. Currently, SLC is predominantly marketed and administered as an oral liquid, which suffers from the disadvantages of inconvenient transport and limited versatility. In this study, we investigated the preparation of direct compression (DC) tablets of SLC. **Methods:** Hydroxypropyl methylcellulose E3 (HPMC E3), polyvinylpyrrolidone K30 (PVP K30), hydroxypropyl cellulose EF (HPC EF), and maltodextrin (MD) were selected as modifying agents; and ammonium bicarbonate (NH_4_HCO_3_) and sodium bicarbonate (NaHCO_3_) were employed as pore-forming agents. Co-spray drying was utilized to prepare 13 kinds of composite particles with different structures. Subsequently, their physical properties and compacting parameters were characterized comprehensively. Finally, the various composite particles were directly compacted into tablets to study the respective effects on the properties of DC tablets. **Results:** The results demonstrated that (i) the SLC composite particles have been successfully produced by co-spray drying, and processing involves physical changes; (ii) the tensile strength (TS) values of PCP-SLC-HPMC-NH_4_HCO_3_, PCP-SLC-PVP-NaHCO_3_, PCP-SLC-HPC-NaHCO_3_, and PCP-SLC-HPMC-NaHCO_3_ were 9.8, 7.2, 8.3, and 7.7 times higher than that of SLC; (iii) all the modifiers studied could improve the DC properties of problematic SLC to some degree, and the combination of HPMC and NH_4_HCO_3_ showed to be the best to markedly improve both the compactibility and flowability of SLC. **Conclusions:** Overall, the design of porous composite particles, composite particles, and porous composite particles in this study successfully produced qualified tablets with high SLC loadings via DC. These findings are favorable for promoting the development and application of natural botanical tablets through DC.

## 1. Introduction

Currently, the exploration of substances of medicine food homology is taking the world by storm, with the search for active ingredients with health or therapeutic properties from natural plants attracting increasing focus [1]. From Shenzhou 10, Shen Ling Cao (SLC) has consecutively accompanied China’s spaceflight, helping Chinese astronauts on their journey of space exploration. As a health product for astronauts, SLC can effectively improve the body’s ability to tolerate hypoxia, increase blood oxygen saturation, relieve symptoms of low-pressure hypoxia, and relieve fatigue. Similarly, SLC has the effects of strengthening immunity, resisting fatigue, resisting hypoxia, and delaying aging as a commercially available product. At present, SLC is mainly available on the market in liquid formulations, such as oral solutions. In contrast to solid dosage forms, liquid preparations tend to have significant drawbacks, including suboptimal stability, challenges with taste masking, and limited portability [2]. Oral solid dosage forms are generally easier to transport and store. They generally do not require the use of a delivery device and allow for less frequent dosing, factors that may contribute to improved medication adherence [3]. Tablets are the most widely used dosage form in the pharmaceutical industry due to their many advantages, including exceptional stability, accurate dosage, ease of use, convenience, cost-effective mass production, and the ability to provide varying drug release patterns [4].

The three primary methods for preparing tablets are dry granulation compression, wet granulation compression, and powder DC [5]. The wet granulation process involves several sequential steps, including sieving, wet massing, granulation (formation of wet granules), drying, and sizing. In contrast, the dry granulation method is less complex, typically consisting of only mixing, sieving, and sizing. Despite these differences, both wet and dry granulation approaches entail relatively intricate production procedures, which contribute to increased time consumption and higher economic costs. Additionally, within the overall tablet manufacturing process, both dry granulation and wet granulation methods generally exhibit lower drug loading capacity [6]. On the other hand, powder direct compression refers to a simplified approach in which the drug powder is sieved and mixed separately with appropriate excipients, followed by DC into tablets without undergoing a granulation step [7]. This streamlined process significantly reduces production complexity, lowers both time and economic costs, and offers considerable economic advantages to pharmaceutical manufacturers. DC is characterized by its simplicity, reduced time and cost demands, suitability for continuous production, and the elimination of issues related to heat and moisture exposure during manufacturing. As a result, it has garnered increasing attention and preference among researchers and pharmaceutical producers [8,9]. However, the successful implementation of the DC process is contingent upon the raw materials possessing specific physic-mechanical properties, including good compressibility and flowability, low hygroscopicity, and minimal sensitivity to lubricants. At present, the majority of active pharmaceutical ingredients (APIs) struggle to meet the necessary requirements for flowability and compressibility due to their inherent physical limitations. Nevertheless, it is difficult for SLC to meet the requirements of DC tableting due to its higher hygroscopicity, viscosity, and poor flowability/compactibility [10]. To facilitate the broader adoption of DC in the production of SLC tablets and to ensure a stable and reliable manufacturing process, numerous researchers are actively engaged in the development of functional excipients or composite materials. Their efforts focus on enhancing the physic-mechanical properties of these materials through synergistic processing strategies, aiming to overcome the inherent limitations of APIs and enable more feasible applications of DC technology [10,11].

Co-processing involves bringing together two or more powders by a common technique, such as co-spray drying, co-fluid-bed, co-freeze-drying co-crystallization, etc., to enhance the functional properties of the powders and mask their undesirable characteristics. This idea relies on interactions at the sub-particle level, enabling the particles of one powder to be integrated into the surface or the interior of particles from another powder [7]. Co-spray drying has been extensively utilized in the materials industry and represents an economical and highly effective technique for transforming liquid substances into dry powders on an industrial scale [12]. This method offers numerous advantages, such as extremely rapid drying (typically completing within about 30 s), the ability to operate at relatively low temperatures, minimal risk of microbial degradation, and the capacity to consistently produce high-quality products without introducing any detrimental effects [13]. When compared to freeze-drying, co-spray drying demonstrates superior cost efficiency [14]. This advantage stems from reduced requirements for storage and transportation, as well as lower operational and maintenance demands. Due to the short residence time of materials within the drying chamber and the swift evaporation of moisture, the process can maintain relatively low particle temperatures. As a result, co-spray drying is particularly well-suited for the drying of heat-sensitive materials, enabling better preservation of both product integrity and final powder quality. Functioning as a one-step continuous process, co-spray drying is capable of producing powders with either fine or coarse agglomerates, all within a relatively narrow and controlled particle size distribution. The resulting particles typically exhibit near-spherical morphology and demonstrate improved compaction properties, which are beneficial for downstream applications such as tableting and encapsulation [15]. Co-spray drying technology is commonly used to enhance both the flowability and compactibility of powders in the pharmaceutical field [16]. As a continuous process, it can be easily automated and is suitable for in-line product analysis, increasing production efficiency. This co-processing technique reduces time to market and improves product consistency, providing significant economic benefits to the pharmaceutical industry [17]. Therefore, co-spray drying was employed as the co-processing method to prepare SLC composite particles in this study. It has provided an effective way to realize direct tablet pressing of SLC powder and solve the above negative effects [18].

Currently, modified particles with different structures, such as porous particles, composite particles, porous composite particles, etc., can be produced through co-spray drying by adding modifiers or pore-forming agents. In our previous reports, we found that composite particles improved the flowability and compactibility of active pharmaceutical ingredients (APIs) [19] and retarded the disintegration behavior of tablets [20]. Furthermore, the porous structure (Ps) of the particles provides good dissolution and release properties due to the large specific surface area (SSA) increasing the probability of drug contact with dissolution media [21]. The majority of Ps are produced using pore-forming agents. Generally used pore-forming agents include sodium bicarbonate (NaHCO_3_) and ammonium bicarbonate (NH_4_HCO_3_), which because of their inherent properties absorb thermal energy and decompose to produce water vapor and carbon dioxide (CO_2_). This results in the dried particles having a porous structure, which increases the compressibility of the drug extract powder and reduces the disintegration time of the tablet [22]. Thus, porous composite particles (PCPs) have been introduced to improve the flowability and compactibility of APIs and the disintegration behavior of tablets produced by DC [17]. In our early reports, hydroxypropyl methylcellulose (HPMC E3), polyvinylpyrrolidone (PVP K30) hydroxypropyl cellulose EF (HPC), and maltodextrin (MD) exhibited great potential in improving the flowability and compactibility of APIs [23,24,25].

In light of the above, this research aimed to (i) prepare composite particles (CPs), porous particles (Ps), and porous composite particles (PCPs) by co-spray drying; (ii) compare the direct compaction properties of SLC of particles with different structures and explore the improving mechanism of flowability and compactibility properties of SLC based on different particle structures; (iii) compare the combination effects of four modifiers, i.e., HPMC, PVP, HPC, MD, and two pore-forming agents, i.e., NH_4_HCO_3_, NaHCO_3_. 

## 2. Results

### 2.1. The Fundamental Physical Properties of the Powder

#### 2.1.1. Analysis of Surface Morphology

**Figure 1:**
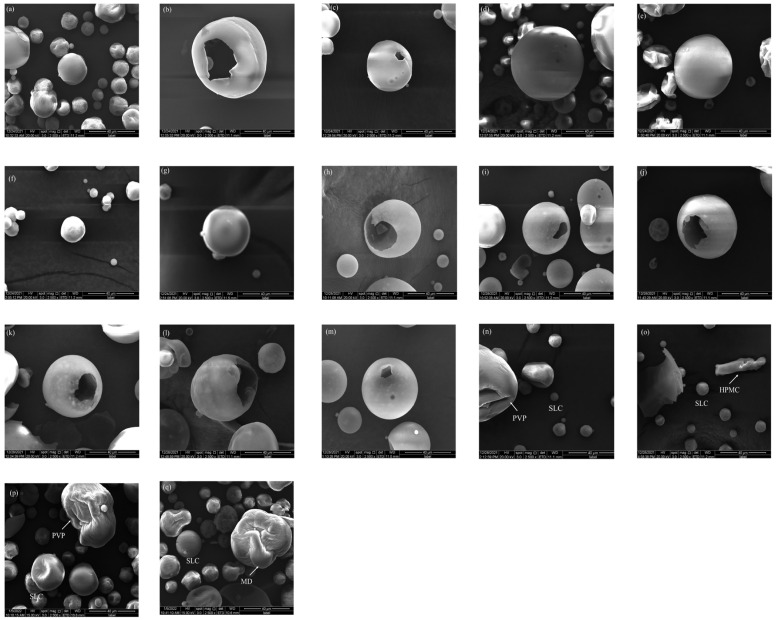
The scanning electron microscope (SEM) of materials (The magnification of the picture is 2500× and the scale bar represents 40 μm). (**a**) SLC; (**b**) P-SLC-NH_4_HCO_3_; (**c**) P-SLC-NaHCO_3_; (**d**) CP-SLC-PVP; (**e**) CP-SLC-HPMC; (**f**) CP-SLC-HPC; (**g**) CP-SLC-MD; (**h**) PCP-SLC-PVP-NH_4_HCO_3_; (**i**) PCP-SLC-PVP-NaHCO_3_; (**j**) PCP-SLC-HPMC-NH_4_HCO_3_; (**k**) PCP-SLC-HPMC-NaHCO_3_; (**l**) PCP-SLC-HPC EF-NH_4_HCO_3_; (**m**) PCP-SLC-HPC EF-NaHCO_3_; (**n**) PM-SLC-PVP; (**o**) PM-SLC-HPMC; (**p**) PM-SLC-HPC EF; (**q**) PM-SLC-MD.

The morphologies of the particles at a magnification of 2500× are shown in Figure 1. Scanning electron microscope (SEM) analyses showed that co-spray drying was successful in the preparation of composite particles with different morphologies. It was obvious that all the particles obtained by co-spray drying showed a smooth surface and a spherical structure with a uniform particle size distribution. Compared with physical mixtures (PMs), all the particles obtained by co-spray drying, i.e., CPs, Ps, and PCPs, exhibited significantly different surface morphology, including the following characteristics: (i) the modifiers and the raw materials were completely integrated, and the original form could not be seen after co-spray drying; (ii) the particle sizes of CPs were bigger than the raw material SLC and showed obvious wrinkles on the particle surface; (iii) all the particles co-spray-dried with a pore-forming agent exhibited an obvious porous structure, i.e., Ps and PCPs. Meanwhile, the particles prepared with NH_4_HCO_3_ showed a greater and larger porous structure than the particles prepared with NaHCO_3_.

#### 2.1.2. FT-IR

**Figure 2:**
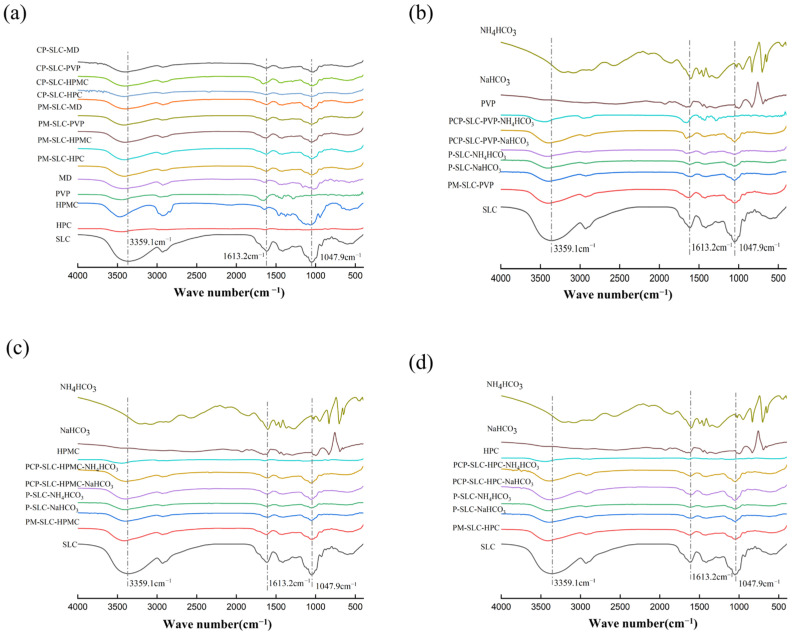
The FT-IR images of SLC materials. (**a**) CP and PM group; (**b**) PVP group; (**c**) HPMC group; (**d**) HPC group.

The FT-IR spectrum of SLC composite particles is presented in Figure 2. The spectral analysis reveals several characteristic peaks corresponding to the functional groups of the constituent polymers. The broad peak observed at 3472 cm^−1^ is attributed to the stretching vibration of the hydroxyl (-OH) group present in HPMC. The peak at 2936 cm^−1^ corresponds to the stretching vibration of the methylene (-CH) groups. Additionally, the stretching vibrations associated with ether bonds and the C-O bonds on the sugar ring are found within the range of 1065 to 1250 cm^−1^ [17]. Regarding the PVP component, its characteristic absorption peaks include the hydroxyl O-H stretching vibration at 3447 cm^−1^, the carbonyl C=O stretching vibration at 1657 cm^−1^, and the C-N stretching vibration at 1292 cm^−1^ [26]. There are no new characteristic peaks in the CPs structure and PM, except for the weakening and migration at 3200 cm^−1^~3500 cm^−1^ and the vibration at 2940 cm^−1^ (-CH) (Figure 2). These observations indicate that the formation of the CPs structure in HPC, HPMC, PVP, and MD as modifiers around SLC does not involve a chemical transformation. There is no formation of new functional groups, it is just a physical change. In the FT-IR of the PCPs samples, apart from the observed weakening and vibration of the characteristic peaks at 3470 cm^−1^ (-OH) and 2940 cm^−1^ (-CH), no new characteristic peaks have emerged. In the FT-IR of P-SLC-NaHCO_3_ and P-SLC-NH_4_HCO_3_ the characteristic peaks of NaHCO_3_ and NH_4_HCO_3_ were not observed, and their peaks are consistent with those of SLC. This indicates that the pore-forming agents were completely decomposed during the co-spray drying process without leaving any residues, and no new functional groups were formed. By means of the PCPs synthesized via co-spray drying, only the modifier constituent was preserved, whereas the pore-forming agent was thermally decomposed and did not persist within the PCP. The process of obtaining composite particles by co-spray drying does not involve any chemical transformation and no new functional groups are created, it is simply a physical modification process.

#### 2.1.3. Thermodynamic Properties

**Figure 3:**
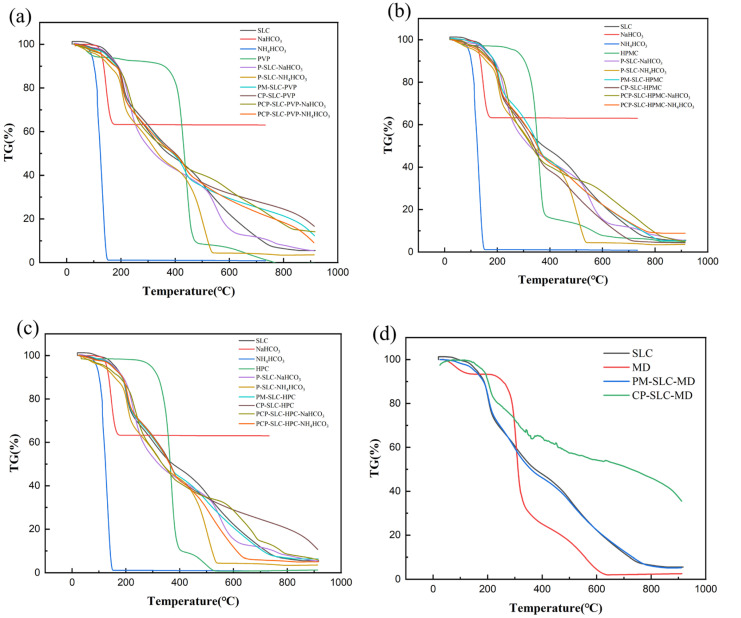
The thermal gravity analysis (TGA) images of SLC materials. (**a**) PVP group; (**b**) HPMC group; (**c**) HPC group; (**d**) MD group.

The results of the thermogravimetric analysis of samples are shown in Figure 3. At 20% weight loss, the temperature of the unmodified sample SLC was 205 °C; the temperatures of the six PCPs were 223 °C (PCP-SLC-HPC-NaHCO_3_), 209 °C (PCP-SLC-HPC- NH_4_HCO_3_), 226 °C (PCP-SLC-HPMC-NaHCO_3_), 207 °C (PCP-SLC-HPMC-NH_4_HCO_3_), 223 °C (PCP-SLC-PVP-NaHCO_3_), and 206 °C (PCP-SLC-PVP- NH_4_HCO_3_), respectively; the temperatures of the four CPs were 206 °C (CP-SLC-HPMC), 208 °C (CP-SLC-HPC), 210 °C (CP-SLC-PVP), and 238 °C (CP-SLC-MD), respectively; the temperatures of the four PMs were all below 210 °C; and the temperatures of the two porous particles were 219 °C (P-SLC-NaHCO_3_) and 201 °C (P-SLC-NH_4_HCO_3_). In summary, it is not difficult to find that the PCPs with NH_4_HCO_3_ as modifier and the CPs with MD as the modifier have higher temperature requirements under the same mass loss conditions.

**Figure 4:**
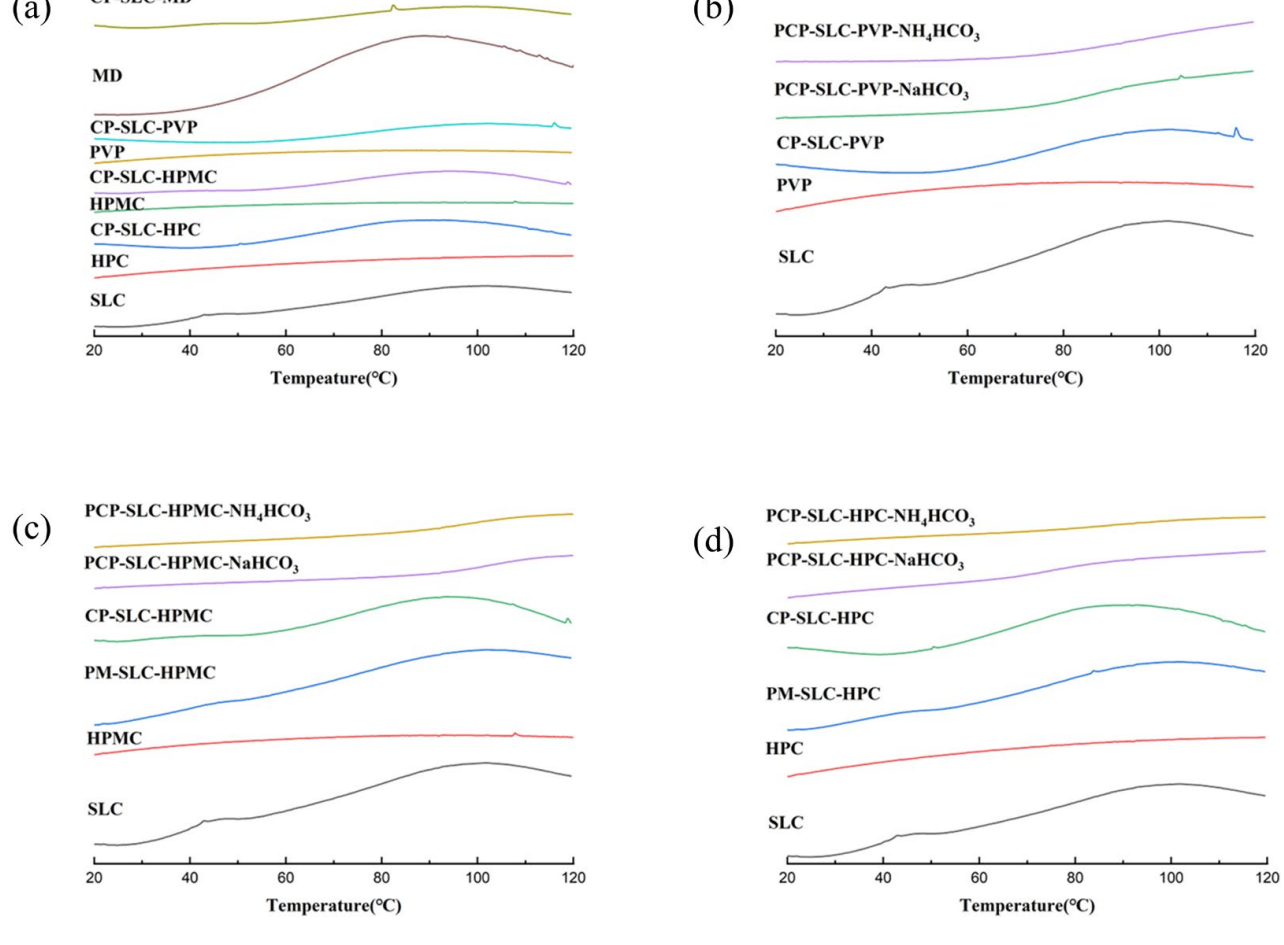
The differential scanning calorimetry (DSC) images of SLC materials. (**a**) CP group; (**b**) PVP group; (**c**) HPMC group; (**d**) HPC group.

This result was again verified in DSC (Figure 4). The PVP shows a higher glass transition temperature (*Tg*) compared to SLC. There is no significant increase in the *Tg* of the CPs, but those with PCPs modified with NH_4_HCO_3_ show an increase in *Tg*. Similarly, HPC and HPMC show similar characteristics. From the figure it can be seen that none of the CPs prepared with modifiers other than MD showed any significant improvement in the *Tg* values of SLC.

The research by Hanna et al. indicated that selecting polymers with a higher *Tg* is advisable during full-scale production to avoid changes in tablet properties [27]. Ioannis Partheniadis et al. found that a higher *Tg* allowed greater plastic deformation of the particles during compaction, thereby increasing the compressibility of the powder [28]. The TGA and DSC experiments of the samples showed that the temperature of PCPs prepared from the modifier HPMC was higher than that of the modifier PVP and HPC at 20% weight loss, and the highest temperature was required for PCP-SLC-HPMC-NH_4_HCO_3_ prepared from the pore-forming agent NH_4_HCO_3_, indicating the best thermodynamic effect. As shown in the figure, the CPs prepared from modifiers other than MD have no significant improvement effects on the *Tg* of SLC. It was shown that the choice of modifiers HPMC, MD, and pore-forming agent NH_4_HCO_3_ improved the compressibility and tablet formability of SLC powders. The use of modifiers prevents changes in the tablet properties of SLC powders during DC. This will provide a reliable research base for later optimization trials and mass production of PCP-SLC and CP-SLC.

#### 2.1.4. Fundamental Properties of Powder

The fundamental physical properties of the SLC powders were studied (Table 1). An appropriate moisture content (MC) facilitates powder compaction, but too high a value can lead to powder caking. This will affect the compaction characteristics of the powder and TS of the tablet [29]. The moisture content of all the materials was between 1.92% and 4.21%. Thus, the influence of moisture content on fundamental and functional properties could be excluded. True density (ρ_true_) serves as a key property of solids, influencing the mixing of powders and the settling of particles in a suspension, as well as influencing various other physical phenomena and processes [30]. The higher the value of ρ_true_, the more voids there are between the particles of the powder or on the surface of the particles. This implies that the powder is fluffier or the particles have a more irregular morphology, which can adversely affect the flowability of the particles and result in a decrease in the powder’s flowability. Composite particles with a PCPs structure have fewer voids between them compared to SLC. The particle size of the powder can influence its flowability. In general, the larger the particle size, the better the flowability. The D_0.5_ of the SLC powder was larger than Ps (13.5 vs. 9.8~10.5 μm) and PCP-SLC-PVP (10.7~10.8 μm). However, the D_0.5_ of the SLC powder was lower than PCP-SLC-HPMC (20.7 μm). The use of HPMC as a modifier to produce PCPs increases particle size and reduces powder porosity to improve the flowability of SLC. Moreover, researchers have found that a more uniform particle size distribution is beneficial in improving the flowability of the powder and reducing the AR [31]. Span refers to the range of particle size distribution. The smaller the span, the narrower the range of powder particles, indicating a more uniform particle size distribution. The span values of the PMs were all higher than those of the unmodified SLC powders, probably due to the incorporation of modifiers. All other composite particles showed improvements, with PCP-SLC-HPMC demonstrating the most significant improvement for SLC in flowability. This result was further confirmed by AR. The porosity of both Ps and PCPs showed an increase compared to SLC powder. In addition, the composite particles using NH_4_HCO_3_ as a pore-forming agent showed an even higher porosity value. This result is compatible with the SEM results in Section 2.1.1.

Overall, the PMs do not improve the flowability of SLC powders. They also reduce the uniformity of the particles, which in turn affects the flowability of the powder. The preparation of PCPs using the co-spray drying technique improves the flowability of SLC powders, with the improvement being more pronounced when HPMC is used as the modifier.

### 2.2. The Functional Properties of Powders

#### 2.2.1. Flowability

Flowability plays a crucial role in the formulation design and processing development stages of DC [32]. Materials with poor flowability can lead to significant variations in tablet weight and uneven distribution of active ingredients within the tablet. Various factors are used to assess the flowability of powders [23] (Table 1). Nevertheless, the complex relationship between internal friction and particle adhesion means that powder flowability cannot be accurately assessed by a solitary value [33]. Various methods have been employed to assess the flow characteristics of particulate materials, including AR, density, and shear tests, among others [34]. The most commonly used measures, CI, HR, and AR, were selected for analysis in this study and lower values of these measures are usually associated with better flowability of the material [35]. In our study, cohesion index (CoI), caking strength (CS), and powder flow stability distribution (PFSD) were also used to comprehensively characterize the flowability of all powders (Table 2). The CS of powders is used to assess the tendency of the powder to change under stress, such as during storage, and the strength of the caking that occurs when the sample is subjected to stress. In this study, the caking strength was used to characterize the tendency of the sample to cake, with higher values indicating greater caking and corresponding to poorer flow properties of the powder. The CoI was used to assess the flowability of the sample, with a lower value indicating a better flowability. Different from the above characters, the PFSD value is equal to 1, showing that the stability of the sample is very good.

The flowability characterization of powders is shown in Table 2. The data showed that the flowability of SLC was not enhanced through physical mixing. The AR for PCPs was the lowest, showing a reduction of 7.38% compared to the unmodified particles. The AR of CPs decreased by 3.7% relative to the unmodified particles, while that of the Ps was the highest, increasing by 1.5% in comparison to the unmodified particles. This may be due to the non-uniform morphology of the PMs, whereas the co-spray-dried particles are spherical (Figure 1). Ps are broken spheres, whereas PCPs have a greater particle size (Table 1) and are an intact porous sphere. Compared with the six PCPs, PCP-SLC-HPMC-NaHCO_3_ showed the smallest AR, CI, HR, and CoI values, which were 37.23, 38.67,1.63, 21.17. And the PFSD value was 0.99. It indicated that PCP-SLC-HPMC-NaHCO3 exhibited the best powder flowability and stability.

Compared with the six PCPs, PCP-SLC-HPMC showed the smallest CS and AR, which were 64.44, 67.40, 40.03, and 37.23. And PCP-SLC-HPMC has a PFSD value that tends to 1.00 (1.05, 0.99). It was found that PCP-SLC-HPMC had the best powder flowability. The values of AR, CoI, and CS for PCP-SLC-HPMC-NH_4_HCO_3_ decreased by 10.27%, 16.15%, and 63.07%, when compared with SLC, respectively. Similarly, compared with SLC, the AR, CI, CS, and CoI values of PCP-SLC-HPMC-NaHCO_3_ decreased by 16.54%, 1.69%, 61.37%, and 21.73%, respectively. The above results were consistent with PCPs flowability characterization results.

In general, PCPs with HPMC tend to resist aggregation and adhesion and they are less prone to clustering, resulting in a slightly improved trend in powder flowability. Previous experiments showed that the NH_4_HCO_3_ had a higher viscosity, which increased with increasing moisture content. Consequently, the use of NH_4_HCO_3_ as a pore-forming agent could result in a fluffier structure and increase the electrostatic adsorption force and van der Waals force between particles, resulting in poor powder flowability. Therefore, NaHCO_3_ has a greater effect as a pore-forming agent than NH_4_HCO_3_ in improving flowability of SLC powders.

#### 2.2.2. Analysis of Compactibility

The ability of a powder to be compressed into a tablet of a predetermined strength under the influence of compression pressure can be referred to as compactibility [36,37]. Poor compressibility can reduce the tensile strength of the tablet, resulting in a tendency to cap and laminate. Generally, at TS 2 MPa, the tablet has good mechanical strength and is suitable for processing and transport [38]. Consequently, the assessment of material compactibility prior to formulation development is critical. In addition, the compression parameters observed during the powder compression process, such as CR, E_2_, Esp, EF, and FES, collectively reflect the compressibility characteristics of the material. The CR is an indicator of the deformation factors of a material during the compression process, and a lower CR indicates an increased compressibility of the material [39]. The values of Esp, which indicate the energy left in the tablet after unloading and are associated with the deformation and binding characteristics of the materials tested, were determined from the work data recorded by the press [40]. A higher ESP value typically indicates that more energy is required to achieve irreversible deformation of the tablet material, which in turn increases the hardness of the tablet and produces a higher TS [23]. The EF is the force required to expel the tablet at the end of compression, with a higher value indicating greater friction between the material and the inner wall during compression [41]. FES is used to characterize the tendency to crack, with higher values indicating greater susceptibility to cracking. Thus, the values for FES and EF should be kept as low as possible in practice.

**Figure 5:**
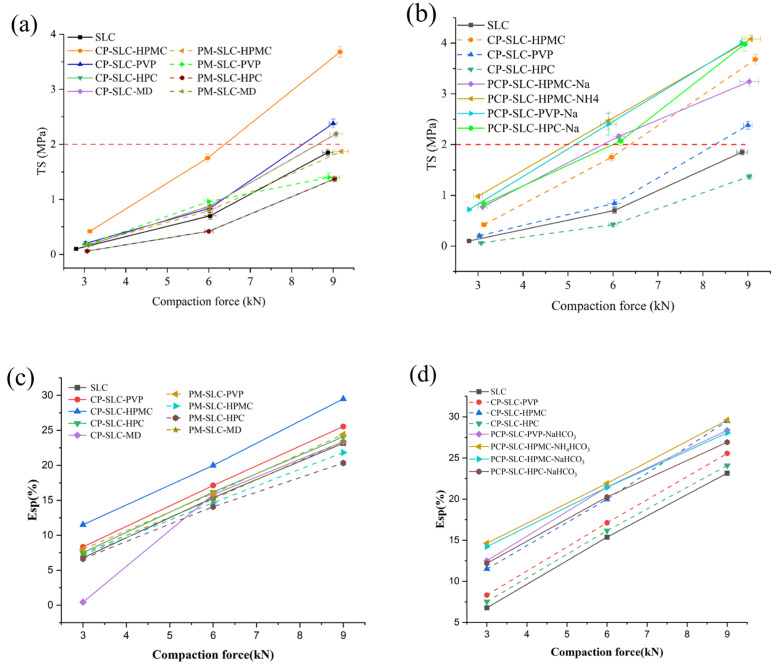
The compactibility characterization of SLC materials. (**a**,**b**) Tensile strength vs. compaction force profiles for SLC; (**c**,**d**) Esp vs. compaction force profiles for SLC.

It can be seen from Figure 5a that at a compaction force of 9 kN, the compaction of the PM is still below 2 MPa and so its compaction is very poor. The compactibility of CPs was better than that of unmodified SLC particles. And the compactibility of CP-SLC-HPMC was better than that of CP-SLC-HPC, CP-SLC-PVP, and CP-SLC-MD. The results indicate that HPMC was more effective than other modifiers for the CPs structure. The four PCPs exhibit TS values in excess of 2 MPa at 6 kN and have a higher Esp compared to CPs and PMs. On the whole, the PCPs have the best compactibility (Figure 5b). PCPs prepared by NH_4_HCO_3_ showed higher TSs than those prepared by NaHCO_3_. The Esp values in Figure 5c,d illustrate the same compression behavior. Among the four PCPs, SLC-HPMC-NH_4_HCO_3_ exhibited the best compactibility, and SLC-HPMC-NaHCO_3_ exhibited the worst (Figure 5b,d). The results showed that when NaHCO_3_ was used as pore-forming agent, the improvement of compactibility by the modifier PVP and HPC were better than that by HPMC. Figure 5b,d reveal that the compactibility of all CPs and PCPs surpasses that of unmodified SLC particles. This improved compressibility is due to the greater plastic deformation ability of the amorphous material compared to the crystalline material. In addition, HPMC, PVP, and HPC modifiers exhibit excellent compressibility. Tableting of the blends showed deformation strongly influenced by HPMC, PVP, and HPC. The plasticity and crush strength of formed tablets were enhanced.

The relevant parameters for all the aforementioned materials during the compression process are presented in Table 3 and Table 4. The data indicate that the CR values for PCPs decreased as the CFs increased, whereas FES, EF, and UDWF all increased as the CF increased. It is shown that the PCPs have better compressibility. Compared to the unmodified SLC particles, the E_2_ value of CPs increases. Similarly, the E_2_ value of PCPs also increases. This indicates that both CPs and PCPs improve the compressibility of SLC powders. The particle structure is a key determinant of the fundamental properties of the powder, with the functional properties of the powder being dictated by these fundamental properties [19]. The reason for CPs showing a higher TS than PMs was that the modifier changed the basic physical characteristic of the particles, for example, ρ_ture_, ρ_b_, ρ_t_, and uniformity were smaller than the unmodified particles. Similarly, the PD and PV of CPs were greater than those of PM and unmodified SLC particles. This indicates that under equivalent mass conditions, CPs occupy a greater volume than the unmodified particles, resulting in larger interparticle gaps. Consequently, CPs are likely to exhibit greater volume reduction and improved compactibility during tableting. The alterations in particle structure led to changes in the powder’s fundamental properties, which in turn affect its functional properties, such as improved powder compressibility [19,42].

In general, it is evident that the particles prepared using NH_4_HCO_3_ exhibit greater TS, SSA, PV, and PD values compared to those prepared with NaHCO_3_. HPMC exhibited superior TS and Esp values compared to HPC and PVP. Consequently, it can be inferred that the compactibility of the materials is significantly influenced by the choice of the pore-forming agent and modifiers. The selection of NH_4_HCO_3_ for preparing Ps is more advantageous for enhancing compactibility than using NaHCO_3_. Moreover, choosing HPMC as a modifier is more conducive to the compactibility than PVP and HPC.

### 2.3. Application of Composite Particles of DC

In this study, the flowability and compactibility properties of different composite particles were compared, from which several points were concluded. First, both parent SLC and PM showed extremely poor DC properties. Second, all CPs and PCPs showed excellent flowability. Third, PCP-SLC-HPMC and CP-SLC-HPMC exhibited both excellent compactibility and flowability simultaneously. These points further confirm that co-spray drying is an effective method to improve the DC properties for problematic SLC powders.

#### 2.3.1. Tablet Structure

**Figure 6:**
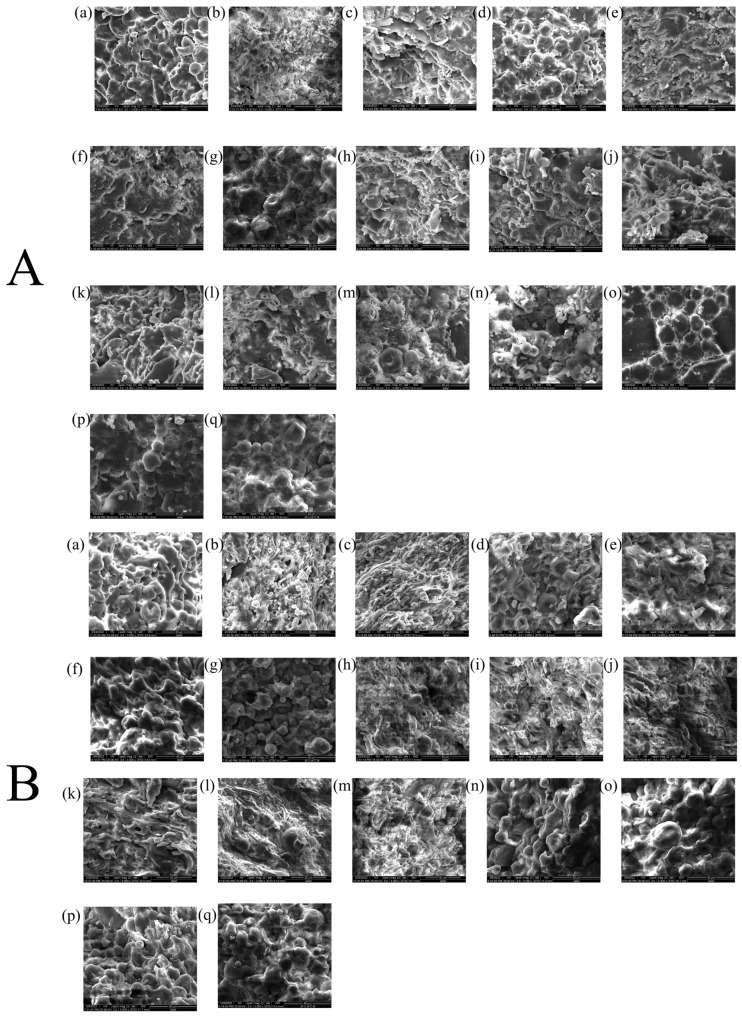
The internal structure of tablets from a scanning electron microscope (Magnification is 3000×, and the scale bar represents 40 μm). (**A**) The cross-section of the tablets; (**B**) the longitudinal section of the tablets: (**a**) SLC; (**b**) P-SLC-NH_4_HCO_3_ (**c**) P-SLC-NaHCO_3_; (**d**) CP-SLC-PVP; (**e**) CP-SLC-HPMC; (**f**) CP-SLC-HPC; (**g**) CP-SLC-MD; (**h**) PCP-SLC-PVP-NH_4_HCO_3_; (**i**) PCP-SLC-PVP-NaHCO_3_; (**j**) PCP-SLC-HPMC-NH_4_HCO_3_; (**k**) PCP-SLC-HPMC-NaHCO_3_; (**l**) PCP-SLC-HPC EF-NH_4_HCO_3_; (**m**) PCP-SLC-HPC EF-NaHCO_3_; (**n**) PM-SLC-PVP; (**o**) PM-SLC-HPMC; (**p**) PM-SLC-HPC EF; (**q**) PM-SLC-MD.

Figure 6 shows the SEM of the cross-section and longitudinal sections of all sample tablets, each with a hardness of 60 N. We compared the micromorphology of the transverse and longitudinal sections of tablets made from Ps and PCPs obtained by the co-spray drying process as well as unmodified SLC particles. It can be found that the tablets made of unmodified SLC particles, both in cross-section and longitudinal section, consist of irregular particles extruded together, and the particles present a kind of adhering and are not close to each other. The limited contact surface between the particles can lead to the formation of loose tablets by DC. The state of the particles in the cross-section and longitudinal section of the tablets made from Ps showed a completely different state compared to the tablets made from unmodified particles. The particles are more prone to fragmentation during compaction due to the formation of Ps during the co-spray drying process. Consequently, more broken particle fragments can be found and the contact area between the particles is greater, resulting in more compacted tablets. Comparing the tablets of the powders prepared with two different pore-forming agents, it can be seen that the tablets of the NH_4_HCO_3_ are more obviously broken and show a more compact state. PCPs have a tablet structure similar to that of Ps due to the incorporation of a pore-forming agent.

The internal structure of the tablets further confirms that the preparation of composite particles by co-spray drying can effectively improve the compressibility of SLC. The Ps structure and PCPs structure can improve the compaction of SLC powders, thereby preventing morphological changes, tablet breakages, and loosening during the tableting process.

#### 2.3.2. Tablet Properties

**Figure 7:**
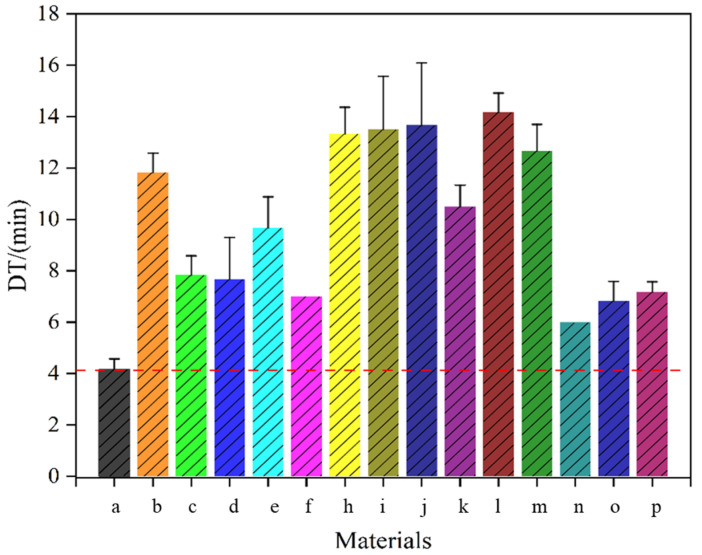
Disintegration times of DC tablets from SLC samples. (a) SLC; (b) P-SLC-NH_4_HCO_3_ (c) P-SLC-NaHCO_3_; (d) CP-SLC-PVP; (e) CP-SLC-HPMC; (f) CP-SLC-HPC; (h) PCP-SLC-PVP-NH_4_HCO_3_; (i) PCP-SLC-PVP-NaHCO_3_; (j) PCP-SLC-HPMC-NH_4_HCO_3_; (k) PCP-SLC-HPMC-NaHCO_3_; (l) PCP-SLC-HPC EF-NH_4_HCO_3_; (m) PCP-SLC-HPC EF-NaHCO_3_; (n) PM-SLC-PVP; (o) PM-SLC-HPMC; (p) PM-SLC-HPC EF.

Disintegration time is a key factor that influences the efficacy of tablet treatments [43]. Thus, it is essential to evaluate these aspects when developing a new tablet formulation. The SLC composite particles prepared by co-spray drying can be subjected to DC tableting without the problem of cap sticking. Figure 7 show the disintegration time of all sample tablets for a hardness of 50 N on tablets of 120 mg. The results show that the disintegration time of DC tablets of all samples is within 20 min, which meets the requirement of the Chinese pharmacopeia (i.e., ≤60 min). The disintegration times of tablets pressed with Ps and PCPs were longer than those of tablets pressed with unmodified particles. The cross-sectional and longitudinal views of the tablets depicted in Figure 6 can explain this phenomenon. The porous structure of the Ps makes the particles more compacted during the tablet pressing process, which in turn slows down the disintegration time of the tablets. Moreover, PCPs are affected not only by the porous structure but also by the modifier, thus delaying the disintegration time of the tablets. The disintegration time of the DC tablets containing PMs, CPs, and PCPs of modifier HPMC was higher than of the DC tablets containing the unmodified particles of SLC. This may be attributed to the inherently high viscosity of HPMC. Studies have shown that the HPMC content affects the viscosity of the medium, thereby slowing disintegration and release [44].

## 3. Discussion

To make natural phytopharmaceuticals more portable and consumable, tablet formulations are unavoidable. The economics of producing large batches of tablets has established DC as the preferred method of tablet production. However, the requirements for powder properties in DC are stringent. In essence, the formulation design for DC is both important and challenging. Therefore, in order to produce a robust tablet, it is essential to have a comprehensive understanding of the material’s properties. Thus, we used co-spray drying to produce SLC particles, Ps, CPs, and PCPs, aiming to explore the unique properties of different powders to enhance the DC performance of SLC.

All the CPs and PCPs exhibited much better flowability and compactibility compared to parent SLC and the corresponding PMs. Different modifiers exhibited significantly different influences on the DC properties of CPs and PCPs. Specifically, PCP-SLC-HPMC-NH_4_HCO_3_ containing both HPMC as a modifier and NH_4_HCO_3_ as a pore-forming agent exhibited the best compactibility. On the other hand, PCP-SLC-HPMC-NaHCO_3_ with HPMC as the modifier and NaHCO_3_ as the pore-forming agent had the best flowability. The improvements depend on the structural integrity and fundamental properties of the composite particles as well as the inherent properties of the modifier, such as compressibility, viscosity, and surface tension. Overall, this study successfully improved SLC (an extracted powder with very poor DC properties) to be directly compressible (SLC-based CPs and PCPs) and prepared qualified tablets with high SLC content via DC. For future research, HPMC could be explored as a potential modifier, and the types and dosages of pore-forming agents could be further refined to develop PCPs with superior flowability and compressibility. These advancements would significantly advance the development and application of SLC-based health products.

## 4. Materials and Methods

### 4.1. Materials

SLC water extract solution was extracted by Jiangzhong Pharmaceutical Co., Ltd. (Nanchang, Jiangxi, China) with an extraction temperature of 95 °C, and the extraction was carried out twice. After that, the water extraction was concentrated by thickener (Jiangzhong Pharmaceutical Co., Ltd., Nanchang, Jiangxi, China). Polyvinylpyrrolidone K30 (PVP K30, Ashland, Wilmington, DL, USA), hydroxypropyl methylcellulose E3 (HPMC E3, Ashland), hydroxypropyl cellulose EF (HPC EF, Ashland), sodium bicarbonate (NaHCO_3_, Sinopharm Chemical Reagent Co., Ltd., Shanghai, China), ammonium bicarbonate (NH_4_HCO_3_, Sinopharm Chemical Reagent Co., Ltd., Shanghai, China), maltodextrin (MD, Hefei Shanhe Medical Technology Co., Ltd., Hefei, China) and magnesium stearate (Mgst, Sinopharm Chemical Reagent Co., Ltd., Shanghai, China).

### 4.2. Preparation of Materials

#### 4.2.1. Preparation of Particles

The unmodified particles and composite particles of SLC were prepared using a Mobile Minor 2000 spray dryer (Niro Company, Slagelse, Denmark) for co-spray drying. The process parameters and conditions were set as follows: 150 °C for inlet temperature, feed pump speed 15 rpm, and atomization pressure 0.26 bar. The relative humidity and temperature in the laboratory during the preparation process were 31–40% and 18–23 °C. The specific proportions of each sample are shown in Table 5.

#### 4.2.2. Physical Mixtures

The four types of PMs, each corresponding to CPs, were prepared by mechanical stirring for 5 min at a rotational speed of 35 rpm using a small-scale Rom min || mixer manufactured by J. Engelsmann Company, Ludwigshafen am Rhein, Germany (Table 5).

### 4.3. Performance of Powder

#### 4.3.1. Moisture Content (MC)

The MC of the materials was characterized by a halogen moisture analyzer (HE53, Mettler Toledo Ltd., Zurich, Switzerland), and the detection temperature was 105 °C. During experimental operation, environmental humidity was controlled to be less than or equal to 45%.

#### 4.3.2. Angle of Repose (AR)

The fixed funnel technique was employed to ascertain the angle of repose (AR) for the sample. Maintaining a constant height, the powdered sample was allowed to flow through a funnel with a fixed internal diameter at a constant rate, eventually forming a symmetrical cone [45]. The fixed base radius (r) and cone height (h) were determined, and were used to calculate AR (θ) for each sample using Equation (1). Each sample was measured three times in parallel.

(1) $$tan\theta =\frac{h}{r}$$

#### 4.3.3. True Density (ρ_ture_)

The ρ_ture_ of the materials was measured using an AccuPyc II 1340 (Micromeritics, Norcross, GA, USA). Before using the instrument, it was properly calibrated. Calibration was performed using a 10 cm^3^ measuring cup to ensure accuracy during testing. Nitrogen gas was used for blowing, and 5 parallel measurements for each sample were performed, with the average value selected.

#### 4.3.4. Density, Carr’s Index (CI), and Hausner Ratio (HR)

The bulk density (ρ_b_) and tapped density (ρ_t_) were determined by a powder characteristic tester (BT-1000, Bettersize Instruments, Ltd., Dandong, China). The CI and HR were calculated based on ρ_b_ and ρ_t_, respectively, using Equations (2) and (3) [46].

(2) $$CI=\frac{\rho_{t}-\rho_{b}}{\rho_{t}}$$

(3) $$HR=\frac{\rho_{t}}{\rho_{b}}$$

#### 4.3.5. Particle Size (D_0.5_), Particle Size Distribution (Span), and Uniformity

The particle size (D_0.5_), particle size distribution (Span), and uniformity were assessed using an Mastersizer 2000 (MS 2000) (Malvern, Great Malvern, UK) laser granularity analyzer. Feeding speed and dispersing air pressure are 43% and 1.65 bar, respectively. Each sample was subjected to three parallel measurements to ensure accuracy.

#### 4.3.6. Surface Morphology

The morphology of particles was examined under a scanning electron microscope (SEM) (Quanta 250, FEI, Medford, MA, USA) at an acceleration voltage of 30 kV. Samples were observed at different magnifications and sputter-coated (Leica EM ACE600, Leica Biosystems, Vienna, Austria) with gold–palladium.

#### 4.3.7. Texture Parameters

The cohesion index (CoI), caking strength (CS), and powder flow stability distribution (PFSD) of the SLC samples were evaluated using a texture analyzer (SMSTA. XT plus, Stable Micro Systems, Godalming, UK). Measurements were performed in triplicate. The testing setup included a specific cylindrical glass container with a height of 120 mm and an inner diameter of 48 mm, a designated rotating spiral blade (rotor number: R48/50/10/2/A), and a force sensing element capable of measuring up to 5.0 kg. Prior to each test, both force and height calibrations were performed to ensure measurement accuracy.

#### 4.3.8. Specific Surface Area (SSA), Pore Volume (PV), and Pore Diameter (PD)

BET specific surface areas (SSAs), pore volumes (PVs), and pore diameters (PDs) of samples were measured by a surface area and pore volume analyzer (TriStar3000, Micromeritics Instrument Corp., Norcross, GA, USA). Nitrogen adsorption isotherms of samples were recorded at the temperature of liquid N_2_. As a result, SSA was calculated using the Brunauer–Emmet–Teller (BET) equations and the Barrett–Joyner–Halenda (BJH) technique, respectively. PV and PD were derived using the BJH method.

#### 4.3.9. Fourier-Transform Infrared Radiation (FT-IR)

The main functional groups of the samples were studied by Fourier-transform infrared radiation (FT-IR) to determine whether new functional groups were formed during the co-processing. The scanning parameters are set as follows: the spectral range is 400–4000 cm^−1^, the resolution is 4 cm^−1^, the number of scans is 16, and a specific point on the sample has been selected for scanning.

#### 4.3.10. Thermogravimetric Analysis (TGA)

A TG/DTA6300 thermogravimetric analyzer (Exstar 6000, Hitachi, Tokyo, Japan) was used to determine the moisture content of the spray-dried powders. Approximately 5 mg of each powder was taken in a crucible, and the particles were subjected to a nitrogen environment with a gas flow rate of 200 mL/min. The scanning was conducted from 25 °C to 1000 °C at a heating speed of 10 °C/min.

#### 4.3.11. Differential Scanning Calorimetry (DSC)

Thermal properties of the SLC composite particles and the physical mixtures of the formulations were examined by a differential scanning calorimeter (Diamond DSC; Perkin-Elmer, Waltham, MA, USA). Nitrogen was used as the protective gas during the heating process, which was conducted at a controlled heating rate of 10 °C per minute, gradually increasing the temperature from 20 °C to 120 °C.

#### 4.3.12. Compactibility

The material is directly compressed into tablets using a rotary tablet press (model TR-D8, manufactured by ERWEKA, Langen, Germany), operating at a compression speed of 10,000 tablets per hour. The compression forces (CFs) applied during the process are set at three different levels: 3 kN, 6 kN, and 9 kN. To avoid sticking and smearing, a blank MgSt should be compacted before each sample is pressed [47]. The tablets’ breaking force (F, N) was assessed promptly utilizing a crushing force tester (YD-20KZ, TIANDA TIANFA, Tianjin, China). The tensile strength (TS, MPa) was determined based on the breaking force (F, N), thickness (T, mm), and diameter (d, mm) using Equation (4), which facilitated a quantitative assessment of the samples’ compressibility within the specified pressure range [48].

(4) $$TS=\frac{2F}{\pi DT}$$

### 4.4. Powder Compaction

#### 4.4.1. Ejection Force and Upper Die Wall Force

Ejection force (EF) and upper die wall force (UDWF) during compression are recorded by the data analysis system of the tablet press.

#### 4.4.2. Power and Energy

The friction energy (E_1_), the energy retained during unloading (E_2_), and the energy loss during unloading (E_3_) are automatically analyzed and recorded by the press analysis system during the compression process. The total energy (E_T_), the percentage of each energy in the total energy (R_i_), the percentage of net energy except friction energy (PL), and the effective compression work net energy per unit of quality (E_SP_) are calculated by the following equations (Equations (5)–(8)):

(5) $$R_{T}=E_{1}+E_{1}+E_{1}$$

(6) $$R_{3}=\frac{E_{3}}{E_{T}}\times 10$$

(7) $$PL=\frac{E_{2}}{E_{2}+E_{3}}$$

(8) $$E_{SP}=\frac{E_{2}}{M}$$

#### 4.4.3. Internal Structural Morphology of the Tablets

A QUANTA FEG 259 scanning electron microscope was used to examine the surface morphology of longitudinal and transverse sections of each tablet sample. Longitudinal sections of each sample were first cut and positioned on a conductive gel surface, followed by gold sputter coating using a Leica EM ACE600 (Leica, Wetzlar, Germany) instrument. Surface morphology observations were made at an accelerating voltage of 30 kV for both the transverse and longitudinal sections of the sample slices.

#### 4.4.4. Tablet Disintegration

The disintegration time of each sample tablet was evaluated according to the protocol for testing the disintegration limits of tablets, as described in the 2020 edition of the Chinese pharmacopeia. The disintegration limit of each sample tablet was recorded using a ZB-1E intelligent disintegration instrument. The cradle method was used for this determination, with pure water as the disintegration medium at a temperature of 37 ± 0.5 °C. Measurements were performed in 6 tablets in parallel.

## Figures and Tables

**Table 1:** The fundamental properties of the particle powders studied in this work (mean ± SD: n = 3).

Sample	MC (%)	ρ_b_ (g/mL)	ρ_t_ (g/mL)	ρ_true_ (g/mL)	D_0.5_ (μm)	Un	Span	BET SSA (m^2^/g)	Ad PV (cm^2^/g)	De PV (cm^2^/g)	Ad PD (nm)	De PD (nm)
a	2.13 ± 0.03	0.3593 ± 0.0006	0.5924 ± 0.0102	1.2913 ± 0.0007	13.4823 ± 0.0321	0.5643 ± 0.0101	1.8263 ± 0.0201	0.3787 ± 0.0017	0.000646	0.000638	87.509	80.362
b	4.21 ± 0.03	0.0627 ± 0.0021	0.0955 ± 0.0040	0.4695 ± 0.0004	10.5050 ± 0.1032	0.6533 ± 0.0025	2.1227 ± 0.0074	1.2402 ± 0.0180	0.002137	0.002187	139.985	116.822
c	3.52 ± 0.03	0.1060 ± 0.0000	0.1614 ± 0.0014	0.5209 ± 0.0004	9.7910 ± 0.0601	0.7103 ± 0.0050	2.2970 ± 0.0125	0.8784 ± 0.0084	0.001395	0.001442	115.137	92.913
d	2.27 ± 0.03	0.2540 ± 0.0021	0.4097 ± 0.0154	1.2411 ± 0.0004	13.8463 ± 0.1276	0.5283 ± 0.0084	1.7200 ± 0.0225	0.2397 ± 0.0090	0.000391	0.000434	311.343	293.510
e	2.43 ± 0.03	0.2487 ± 0.0021	0.4060 ± 0.0215	1.1049 ± 0.0008	24.5400 ± 0.0390	0.9643 ± 0.0206	2.1557 ± 0.0290	0.3558 ± 0.0035	0.000644	0.000655	113.780	107.683
f	2.28 ± 0.03	0.3893 ± 0.0102	0.6420 ± 0.0261	1.2678 ± 0.0010	14.9400 ± 0.0269	0.5300 ± 0.0087	1.7093 ± 0.0193	0.2134 ± 0.0042	0.000380	0.000387	204.497	155.211
g	2.28 ± 0.03	0.3503 ± 0.0091	0.5531 ± 0.0098	1.2795 ± 0.0007	13.4347 ± 0.0343	0.5653 ± 0.0118	1.8277 ± 0.0327	0.3686 ± 0.0029	0.000674	0.000679	108.541	101.777
h	3.45 ± 0.01	0.1177 ± 0.0012	0.1919 ± 0.0056	0.6073 ± 0.0004	10.7367 ± 0.1069	0.6987 ± 0.0049	2.2733 ± 0.017	0.4460 ± 0.0210	0.000884	0.000885	381.459	314.406
i	3.03 ± 0.06	0.1427 ± 0.0060	0.2278 ± 0.0132	0.7217 ± 0.0008	10.7863 ± 0.0536	0.7100 ± 0.0148	2.3117 ± 0.0384	0.5263 ± 0.0157	0.000761	0.000789	248.231	277.170
j	3.62 ± 0.03	0.1360 ± 0.0020	0.2267 ± 0.0033	0.9326 ± 0.0011	20.7403 ± 0.0299	1.0767 ± 0.0462	2.8067 ± 0.0319	0.6037 ± 0.0281	0.000773	0.000824	223.533	118.470
k	3.28 ± 0.03	0.1463 ± 0.0021	0.2386 ± 0.0015	1.0731 ± 0.0016	20.7137 ± 0.0279	0.7593 ± 0.0057	2.4423 ± 0.0193	0.5476 ± 0.0125	0.000770	0.000783	156.582	126.144
l	2.82 ± 0.03	0.1057 ± 0.0032	0.1585 ± 0.0055	0.5156 ± 0.0004	13.0220 ± 0.0285	0.7210 ± 0.0151	2.3127 ± 0.0402	0.3854 ± 0.0225	0.000779	0.000780	372.150	339.027
m	2.80 ± 0.05	0.1450 ± 0.0010	0.2132 ± 0.0015	0.6114 ± 0.0004	13.8087 ± 0.0375	0.8493 ± 0.1166	2.5083 ± 0.0847	0.4423 ± 0.0129	0.000736	0.000730	200.719	160.132
n	1.93 ± 0.03	0.4207 ± 0.0021	0.6164 ± 0.0226	1.2894 ± 0.0006	15.1680 ± 0.0795	0.9513 ± 0.0087	2.7570 ± 0.0286	0.2918 ± 0.0026	0.000504	0.000528	110.382	95.244
o	1.92 ± 0.03	0.4350 ± 0.0044	0.6275 ± 0.0084	1.3079 ± 0.0004	14.8217 ± 0.0656	0.9900 ± 0.0260	2.8560 ± 0.0387	0.3381 ± 0.0023	0.000496	0.000502	82.100	77.795
p	1.78 ± 0.03	0.4670 ± 0.0108	0.6579 ± 0.0201	1.3190 ± 0.0008	16.3103 ± 0.2599	3.7100 ± 0.3822	17.8927 ± 1.9880	0.3147 ± 0.0044	0.000379	0.000413	90.643	80.956
q	2.03 ± 0.03	0.4247 ± 0.0057	0.6067 ± 0.0081	1.3137 ± 0.0002	14.2833 ± 0.1553	0.6590 ± 0.0340	2.0530 ± 0.0544	0.3287 ± 0.0026	0.000457	0.000457	81.255	75.557

MC: moisture content; ρ_true_: true density; ρ_b_: bulk density; ρ_t_: tapped density; D_0.5_: median particle size; Un: uniformity; Span: particle size distribution; BET SSA: BET specific surface area; Ad PV: BJH adsorption cumulative volume of pores between 17.000 and 3000.00 nm diameter cumulative adsorption pore volume; De PV: BJH desorption cumulative volume of pores between 17.000 and 3000.00 nm diameter cumulative desorption pore volume; Ad PD: BJH adsorption average pore diameter (4 V/A): adsorption pore size; De PD: BJH desorption average pore diameter (4 V/A): desorption pore size.

**Table 2:** Particle powder flowability properties based on co-spray drying and physical mixing preparation (mean ± SD: n = 3).

Sample	AR (°)	CI	HR	CoI	Cs	PFSD
a	44.61 ± 0.68	39.33 ± 1.15	1.65 ± 0.03	27.05 ± 1.55	174.48 ± 8.31	0.79 ± 0.05
b	46.12 ± 0.00	34.33 ± 0.58	1.52 ± 0.01	13.34 ± 0.89	118.27 ± 7.61	1.43 ± 0.09
c	45.37 ± 0.65	34.33 ± 0.58	1.52 ± 0.01	18.51 ± 0.36	72.67 ± 2.75	1.20 ± 0.01
d	40.91 ± 0.76	38.00 ± 0.00	1.61 ± 0.00	17.77 ± 0.32	164.69 ± 4.91	0.93 ± 0.05
e	40.91 ± 0.76	38.67 ± 2.31	1.63 ± 0.06	28.16 ± 0.42	179.79 ± 6.58	0.94 ± 0.07
f	42.61 ± 0.00	39.33 ± 1.15	1.65 ± 0.03	31.26 ± 0.69	175.8 ± 5.15	0.96 ± 0.09
g	40.91 ± 0.76	36.67 ± 0.58	1.58 ± 0.01	19.01 ± 1.23	167.03 ± 1.63	0.93 ± 0.07
h	43.02 ± 0.70	38.67 ± 1.15	1.63 ± 0.03	12.47 ± 0.97	92.73 ± 6.35	1.28 ± 0.08
i	42.61 ± 0.00	37.33 ± 1.15	1.60 ± 0.03	15.87 ± 0.47	78.17 ± 0.83	1.13 ± 0.09
j	40.03 ± 0.00	40.00 ± 0.00	1.67 ± 0.00	22.68 ± 0.89	64.44 ± 2.37	1.05 ± 0.07
k	37.23 ± 0.00	38.67 ± 1.15	1.63 ± 0.03	21.17 ± 0.70	67.40 ± 4.60	0.99 ± 0.05
l	42.61 ± 0.00	33.33 ± 0.58	1.50 ± 0.01	13.39 ± 1.07	82.61 ± 11.09	1.04 ± 0.07
m	41.35 ± 0.00	32.00 ± 0.00	1.47 ± 0.00	12.70 ± 0.70	59.76 ± 4.29	1.06 ± 0.06
n	44.61 ± 0.68	31.67 ± 3.79	1.47 ± 0.08	30.36 ± 0.90	200.97 ± 6.86	0.97 ± 0.08
o	43.83 ± 0.00	30.67 ± 1.15	1.44 ± 0.02	36.33 ± 0.76	183.11 ± 5.74	1.04 ± 0.06
p	39.57 ± 0.79	29.00 ± 1.00	1.41 ± 0.02	19.09 ± 0.79	188.21 ± 9.41	1.07 ± 0.04
q	43.83 ± 0.00	30.00 ± 0.00	1.43 ± 0.00	35.25 ± 1.54	158.43 ± 3.84	1.13 ± 0.05

AR: angle of repose; CI: Carr’s index; HR: Hausner ratio; CoI: cohesive index; adhesion index; Cs: cake strength; PFSD: powder flow stability distribution.

**Table 3:** Summary of compression process parameters of SLC-1 (Mean ± SD: n = 3).

Materials	CF (kN)	CR (%)	FES (%)	EF (N)	UDWF (N)
a	2.80 ± 0.00	29.771 ± 0.010	5.104 ± 0.101	13.517 ± 0.557	0.307 ± 0.008
	6.03 ± 0.15	25.625 ± 0.030	6.981 ± 0.125	86.589 ± 3.729	0.638 ± 0.015
	8.87 ± 0.12	23.208 ± 0.062	9.594 ± 0.400	126.302 ± 3.139	0.976 ± 0.022
d	3.03 ± 0.06	30.025 ± 0.033	5.138 ± 0.332	31.413 ± 1.715	0.328 ± 0.008
	6.03 ± 0.15	26.039 ± 0.030	7.616 ± 0.240	87.240 ± 2.033	0.648 ± 0.010
	9.00 ± 0.10	23.322 ± 0.025	9.629 ± 0.618	117.350 ± 2.689	1.005 ± 0.016
e	3.13 ± 0.06	26.739 ± 0.000	5.391 ± 0.112	16.276 ± 1.849	0.545 ± 0.013
	5.97 ± 0.06	23.067 ± 0.045	7.623 ± 0.202	72.428 ± 5.175	1.120 ± 0.032
	9.17 ± 0.06	20.605 ± 0.058	10.207 ± 0.429	107.910 ± 1.292	2.104 ± 0.074
f	3.07 ± 0.06	29.225 ± 0.033	5.478 ± 0.330	3.287 ± 0.536	0.332 ± 0.006
	6.00 ± 0.10	25.263 ± 0.025	7.447 ± 0.234	46.224 ± 0.746	0.670 ± 0.013
	9.03 ± 0.06	22.923 ± 0.025	10.295 ± 0.367	63.965 ± 1.292	1.050 ± 0.009
g	3.10 ± 0.10	29.149 ± 0.057	4.966 ± 0.235	0.336 ± 0.004	52.450 ± 0.620
	6.03 ± 0.15	25.154 ± 0.033	7.167 ± 0.327	90.332 ± 1.953	0.633 ± 0.022
	9.07 ± 0.15	22.891 ± 0.008	9.739 ± 0.499	123.861 ± 5.221	0.962 ± 0.039
i	2.80 ± 0.00	15.516 ± 0.072	7.171 ± 0.511	74.577 ± 1.605	0.516 ± 0.002
	5.90 ± 0.17	13.178 ± 0.050	11.231 ± 0.417	155.111 ± 40.316	1.024 ± 0.020
	8.87 ± 0.06	11.559 ± 0.025	15.354 ± 0.459	253.223 ± 9.601	1.129 ± 0.029
j	3.00 ± 0.10	15.250 ± 0.042	7.439 ± 0.300	71.289 ± 4.709	0.574 ± 0.026
	5.90 ± 0.10	12.845 ± 0.053	12.286 ± 0.693	92.285 ± 1.692	0.967 ± 0.066
	9.07 ± 0.21	11.576 ± 0.027	15.189 ± 0.209	116.374 ± 3.251	1.351 ± 0.029
k	3.10 ± 0.10	15.230 ± 0.027	7.748 ± 0.203	30.468 ± 1.947	0.621 ± 0.013
	6.13 ± 0.12	12.934 ± 0.057	12.205 ± 0.501	72.103 ± 4.793	1.305 ± 0.048
	9.03 ± 0.21	11.669 ± 0.003	16.371 ± 0.652	97.819 ± 1.492	1.830 ± 0.070
m	3.13 ± 0.12	15.118 ± 0.000	6.920 ± 0.013	37.936 ± 3.968	0.509 ± 0.012
	6.17 ± 0.06	13.502 ± 0.073	9.355 ± 0.209	75.988 ± 1.773	0.975 ± 0.010
	8.93 ± 0.06	11.669 ± 0.003	15.539 ± 0.230	87.24 ± 5.356	1.653 ± 0.028
n	3.07 ± 0.12	34.583 ± 0.124	5.694 ± 0.190	19.857 ± 1.715	0.117 ± 0.002
	6.00 ± 0.10	29.868 ± 0.094	7.527 ± 0.329	68.522 ± 1.491	0.217 ± 0.010
	8.90 ± 0.26	27.107 ± 0.113	10.187 ± 0.512	106.283 ± 5.378	0.321 ± 0.006
o	3.07 ± 0.06	33.565 ± 0.045	5.286 ± 0.125	24.251 ± 1.848	0.116 ± 0.001
	6.00 ± 0.10	29.390 ± 0.039	7.859 ± 0.330	69.820 ± 1.459	0.218 ± 0.006
	9.20 ± 0.17	26.511 ± 0.130	10.960 ± 0.667	97.332 ± 1.972	0.327 ± 0.010
p	3.00 ± 0.00	33.587 ± 0.039	5.617 ± 0.195	18.858 ± 0.807	0.122 ± 0.001
	6.00 ± 0.10	29.435 ± 0.000	8.235 ± 0.000	54.525 ± 1.715	0.235 ± 0.007
	8.97 ± 0.21	26.713 ± 0.035	10.65 ± 0.513	78.125 ± 3.686	0.334 ± 0.003
q	3.03 ± 0.12	34.007 ± 0.248	5.749 ± 0.362	23.866 ± 1.861	0.122 ± 0.000
	5.90 ± 0.10	28.249 ± 2.305	7.894 ± 0.583	69.823 ± 5.755	0.215 ± 0.002
	8.97 ± 0.21	26.455 ± 0.029	10.438 ± 0.449	108.399 ± 4.257	0.311 ± 0.006

CF: compaction force; CR: compaction ratio; FES: fast elastic stretch; EF: ejection force; UDWF: upper die wall force.

**Table 4:** Summary of compression process parameters of SLC-2 (Mean ± SD: n = 3).

Materials	CF (kN)	E_1_ (Nm)	E_2_ (Nm)	E_3_ (Nm)	ET (Nm)	R_1_ (%)	R_2_ (%)	R_3_ (%)	PL (%)
a	2.80 ± 0.00	1.417 ± 0.067	1.357 ± 0.032	0.077 ± 0.006	2.850 ± 0.104	49.695 ± 0.529	47.618 ± 0.636	2.687 ± 0.107	0.947 ± 0.003
	6.03 ± 0.15	3.087 ± 0.196	3.073 ± 0.090	0.267 ± 0.032	6.427 ± 0.290	48.003 ± 0.957	47.844 ± 0.764	4.153 ± 0.509	0.920 ± 0.009
	8.87 ± 0.12	4.503 ± 0.071	4.630 ± 0.087	0.553 ± 0.081	9.687 ± 0.181	46.495 ± 0.670	47.802 ± 0.762	5.703 ± 0.737	0.893 ± 0.013
d	3.03 ± 0.06	1.700 ± 0.030	1.667 ± 0.047	0.107 ± 0.031	3.473 ± 0.067	48.947 ± 0.541	47.980 ± 0.544	3.073 ± 0.897	0.940 ± 0.017
	6.03 ± 0.15	3.500 ± 0.082	3.423 ± 0.035	0.297 ± 0.006	7.220 ± 0.118	48.473 ± 0.340	47.418 ± 0.298	4.109 ± 0.093	0.920 ± 0.002
	9.00 ± 0.10	5.177 ± 0.146	5.110 ± 0.062	0.573 ± 0.081	10.860 ± 0.288	47.666 ± 0.090	47.065 ± 0.670	5.269 ± 0.601	0.899 ± 0.012
e	3.13 ± 0.06	3.550 ± 0.123	2.303 ± 0.060	0.073 ± 0.006	5.927 ± 0.145	59.891 ± 0.885	38.869 ± 0.876	1.239 ± 0.126	0.969 ± 0.003
	5.97 ± 0.06	6.003 ± 0.055	3.997 ± 0.067	0.303 ± 0.080	10.303 ± 0.091	58.266 ± 0.135	38.794 ± 0.850	2.940 ± 0.760	0.930 ± 0.018
	9.17 ± 0.06	8.633 ± 0.135	5.900 ± 0.010	0.493 ± 0.015	15.027 ± 0.14	57.451 ± 0.363	39.266 ± 0.405	3.283 ± 0.083	0.923 ± 0.002
f	3.07 ± 0.06	1.193 ± 0.058	1.507 ± 0.035	0.120 ± 0.017	2.820 ± 0.085	42.310 ± 1.239	53.444 ± 1.225	4.246 ± 0.508	0.926 ± 0.009
	6.00 ± 0.10	2.547 ± 0.051	3.233 ± 0.071	0.290 ± 0.010	6.070 ± 0.115	41.955 ± 0.253	53.266 ± 0.182	4.780 ± 0.222	0.918 ± 0.004
	9.03 ± 0.06	4.010 ± 0.111	4.817 ± 0.067	0.613 ± 0.040	9.440 ± 0.130	42.477 ± 0.933	51.026 ± 0.570	6.497 ± 0.403	0.887 ± 0.005
g	3.10 ± 0.10	1.750 ± 0.075	0.087 ± 0.006	2.836 ± 0.065	4.673 ± 0.113	37.445 ± 0.995	1.854 ± 0.107	60.701 ± 1.099	0.030 ± 0.002
	6.03 ± 0.15	3.253 ± 0.198	3.160 ± 0.092	0.353 ± 0.025	6.767 ± 0.264	48.051 ± 1.056	46.712 ± 0.474	5.237 ± 0.582	0.899 ± 0.009
	9.07 ± 0.15	4.723 ± 0.035	4.677 ± 0.190	0.627 ± 0.050	10.027 ± 0.145	47.116 ± 0.880	46.630 ± 1.227	6.254 ± 0.567	0.882 ± 0.012
i	2.80 ± 0.00	5.347 ± 0.295	1.500 ± 0.010	0.053 ± 0.012	6.900 ± 0.276	77.457 ± 1.155	21.765 ± 0.962	0.778 ± 0.194	0.966 ± 0.007
	5.90 ± 0.17	6.647 ± 0.170	2.577 ± 0.051	0.290 ± 0.052	9.513 ± 0.180	69.863 ± 0.852	27.094 ± 0.865	3.043 ± 0.493	0.899 ± 0.016
	8.87 ± 0.06	7.287 ± 0.115	3.403 ± 0.055	0.707 ± 0.050	11.397 ± 0.116	63.934 ± 0.368	29.867 ± 0.743	6.198 ± 0.386	0.828 ± 0.012
j	3.00 ± 0.10	5.637 ± 0.442	1.757 ± 0.061	0.190 ± 0.147	7.583 ± 0.639	74.353 ± 0.415	23.233 ± 1.271	2.415 ± 1.663	0.907 ± 0.063
	5.90 ± 0.10	7.097 ± 0.076	2.630 ± 0.072	0.290 ± 0.061	10.017 ± 0.18	70.857 ± 0.832	26.253 ± 0.274	2.890 ± 0.559	0.901 ± 0.016
	9.07 ± 0.21	8.447 ± 0.194	3.553 ± 0.076	0.583 ± 0.110	12.583 ± 0.11	67.120 ± 0.963	28.241 ± 0.750	4.638 ± 0.891	0.859 ± 0.024
k	3.10 ± 0.10	5.070 ± 0.231	1.707 ± 0.061	0.120 ± 0.010	6.897 ± 0.251	73.500 ± 0.968	24.755 ± 0.822	1.745 ± 0.205	0.934 ± 0.006
	6.13 ± 0.12	6.647 ± 0.215	2.573 ± 0.055	0.347 ± 0.045	9.567 ± 0.244	69.471 ± 0.691	26.901 ± 0.186	3.628 ± 0.507	0.881 ± 0.014
	9.03 ± 0.21	7.943 ± 0.228	3.367 ± 0.095	0.637 ± 0.081	11.947 ± 0.235	66.483 ± 0.657	28.178 ± 0.311	5.339 ± 0.779	0.841 ± 0.021
l	3.13 ± 0.12	5.580 ± 0.190	1.463 ± 0.015	0.083 ± 0.032	7.127 ± 0.214	78.292 ± 0.459	20.547 ± 0.713	1.162 ± 0.411	0.946 ± 0.020
	6.17 ± 0.06	6.397 ± 0.567	2.430 ± 0.229	0.230 ± 0.061	9.057 ± 0.723	70.606 ± 1.650	26.866 ± 2.239	2.528 ± 0.591	0.913 ± 0.024
	8.93 ± 0.06	6.377 ± 0.220	3.230 ± 0.026	0.557 ± 0.049	10.163 ± 0.248	62.732 ± 0.638	31.792 ± 0.738	5.476 ± 0.448	0.853 ± 0.012
n	3.07 ± 0.12	1.307 ± 0.015	1.600 ± 0.082	0.117 ± 0.015	3.023 ± 0.076	43.237 ± 1.153	52.898 ± 1.384	3.865 ± 0.553	0.932 ± 0.010
	6.00 ± 0.10	2.490 ± 0.061	3.193 ± 0.070	0.283 ± 0.023	5.967 ± 0.078	41.730 ± 0.695	53.518 ± 0.785	4.752 ± 0.450	0.918 ± 0.008
	8.90 ± 0.26	4.030 ± 0.139	4.887 ± 0.021	0.550 ± 0.066	9.467 ± 0.08	42.566 ± 1.188	51.623 ± 0.608	5.811 ± 0.713	0.899 ± 0.011
o	3.07 ± 0.06	1.360 ± 0.165	1.437 ± 0.023	0.127 ± 0.006	2.923 ± 0.179	46.414 ± 2.778	49.247 ± 2.584	4.339 ± 0.228	0.919 ± 0.002
	6.00 ± 0.10	2.707 ± 0.071	2.930 ± 0.082	0.360 ± 0.017	5.997 ± 0.135	45.134 ± 0.231	48.857 ± 0.386	6.009 ± 0.407	0.89 ± 0.007
	9.20 ± 0.17	4.037 ± 0.211	4.367 ± 0.175	0.680 ± 0.050	9.083 ± 0.355	44.427 ± 0.724	48.072 ± 0.069	7.501 ± 0.727	0.865 ± 0.011
p	3.00 ± 0.00	1.140 ± 0.036	1.327 ± 0.006	0.127 ± 0.012	2.593 ± 0.042	43.952 ± 0.682	51.168 ± 1.031	4.881 ± 0.366	0.913 ± 0.008
	6.00 ± 0.10	2.567 ± 0.074	2.817 ± 0.047	0.357 ± 0.059	5.740 ± 0.142	44.714 ± 0.531	49.078 ± 0.577	6.207 ± 0.961	0.888 ± 0.016
	8.97 ± 0.21	3.817 ± 0.084	4.067 ± 0.093	0.587 ± 0.060	8.470 ± 0.061	45.067 ± 1.306	48.009 ± 0.759	6.924 ± 0.667	0.874 ± 0.009
q	3.03 ± 0.12	1.293 ± 0.029	1.477 ± 0.015	0.090 ± 0.040	2.860 ± 0.026	45.222 ± 0.963	51.633 ± 0.447	3.145 ± 1.401	0.943 ± 0.025
	5.90 ± 0.10	2.500 ± 0.020	3.037 ± 0.081	0.317 ± 0.064	5.853 ± 0.156	42.725 ± 0.798	51.880 ± 0.377	5.395 ± 0.940	0.906 ± 0.015
	8.97 ± 0.21	3.893 ± 0.064	4.683 ± 0.211	0.617 ± 0.038	9.193 ± 0.293	42.365 ± 0.825	50.928 ± 0.681	6.706 ± 0.299	0.884 ± 0.004

CF: compaction ratio; E_1_: friction energy; E_2_: energy retained during unloading; E_3_: energy loss during unloading; ET: total energy; R_1_: percentage of E_1_; R_2_: percentage of E_2_; R_3_: percentage of E_3_; PL: percentage of net energy except friction energy.

**Table 5:** Preparation of combined co-spray drying solutions and characterization of liquid properties.

Material	Sample	SLC (*w*/*w*)	Modifiers (*w*/*w*)	Pore-Forming Agent (*w*/*w*)	Viscosity (mPa·s)	Surface Tension (mN/m)	Solids Content (%)
SLC	a	100	/	/	21.4 ± 0.173	40.7 ± 0.1	0.2378 ± 0.0001
P-SLC-NH_4_HCO_3_	b	100	/	NH_4_HCO_3_-7	14.7 ± 0.252	40.1 ± 0.2	0.1691 ± 0.0042
P-SLC-NaHCO_3_	c	100	/	NaHCO_3_-7	11.7 ± 0.000	40.5 ± 0.1	0.1710 ± 0.0002
CP-SLC-PVP	d	90	PVP-10	/	11.5 ± 0.458	38.1 ± 0.2	0.1488 ± 0.0013
CP-SLC-HPMC	e	90	HPMC-10	/	15.8 ± 0.173	37.6 ± 0.1	0.1096 ± 0.0003
CP-SLC-HPC EF	f	90	HPC EF-10	/	16.2 ± 0.300	38.7 ± 0.1	0.0876 ± 0.0007
CP-SLC-MD	g	90	MD-10	/	13.5 ± 0.000	37.4 ± 0.1	0.1803 ± 0.0003
PCP-SLC-PVP-NH_4_HCO_3_	h	90	PVP-10	NH_4_HCO_3_-7	18.8 ± 0.173	40.1 ± 0.1	0.0833 ± 0.0011
PCP-SLC-PVP-NaHCO_3_	i	90	PVP-10	NaHCO_3_-7	21.4 ± 0.173	39.4 ± 0.1	0.0823 ± 0.0005
PCP-SLC-HPMC-NH_4_HCO_3_	j	90	HPMC-10	NH_4_HCO_3_-7	17.5 ± 0.173	40.0 ± 0.1	0.0717 ± 0.0008
PCP-SLC-HPMC-NaHCO_3_	k	90	HPMC-10	NaHCO_3_-7	21.2 ± 0.173	37.6 ± 0.2	0.0727 ± 0.0003
PCP-SLC-HPC EF-NH_4_HCO_3_	l	90	HPC EF-10	NH_4_HCO_3_-7	22.6 ± 0.173	35.7 ± 0.1	0.0667 ± 0.0002
PCP-SLC-HPC EF-NaHCO_3_	m	90	HPC EF-10	NaHCO_3_-7	15.7 ± 0.173	39.3 ± 0.2	0.0756 ± 0.0003
PM-SLC-PVP	n	90	PVP-10	/	/	/	/
PM-SLC-HPMC	o	90	HPMC-10	/	/	/	/
PM-SLC-HPC EF	p	90	HPC EF-10	/	/	/	/
PM-SLC-MD	q	90	MD-10	/	/	/	/

SLC: Shen Ling Cao powder; CP: core–shell composite particles; P: porous composite particles; PCP: porous core–shell composite particles; HPMC: hydroxypropyl methylcellulose; PVP: polyvinylpyrrolidone; MD: maltodextrin; HPC: hydroxypropyl cellulose; NH_4_HCO_3:_ ammonium bicarbonate; NaHCO_3_: sodium bicarbonate.

## Data Availability

Data presented in this study is contained within the article.

## Abbreviations

The following abbreviations are used in this manuscript:

SLC	Sen Ling Cao
MC	Moisture Content
TS	Tensile strength
CPs	Core–shell particles
PMs	Physical mixtures
Ps	Porous particles
PCPs	Porous core–shell composite particles
DC	Direct compression
NaHCO_3_	Sodium bicarbonate
NH_4_HCO_3_	Ammonium bicarbonate
MD	Maltodextrin
HPC EF	Hydroxypropyl cellulose EF
Mgst	Magnesium stearate
HPMC E3	Hydroxypropyl methylcellulose E3
PVP K30	Polyvinylpyrrolidone K30
